# Supplementary material for: Development of a computer-aided design software for the quantitative evaluation of aesthetic damage
Source: PLoS One. 2019 Dec 18;14(12):e0226322. doi: 10.1371/journal.pone.0226322 (PMC6919621; doi:10.1371/journal.pone.0226322)
Supplement: S1 Code site — 1. (ZIP) [file pone.0226322.s001.zip › [EESC_jr][Projeto]Relatorio_07_21/reports/templates_zip/gentelella-master.zip!/gentelella-master/vendors/DateJS/reports/lcov-report/core/parsing_operators.js.html]

Code coverage report for core/parsing\_operators.js


# Code coverage report for core/parsing\_operators.js

## Statements: 72.12% (150 / 208)      Branches: 65% (52 / 80)      Functions: 70.45% (31 / 44)      Lines: 72.12% (150 / 208)      Ignored: none

All files » core/ » parsing\_operators.js

```
|  |  |  |
| --- | --- | --- |
| 1 2 3 4 5 6 7 8 9 10 11 12 13 14 15 16 17 18 19 20 21 22 23 24 25 26 27 28 29 30 31 32 33 34 35 36 37 38 39 40 41 42 43 44 45 46 47 48 49 50 51 52 53 54 55 56 57 58 59 60 61 62 63 64 65 66 67 68 69 70 71 72 73 74 75 76 77 78 79 80 81 82 83 84 85 86 87 88 89 90 91 92 93 94 95 96 97 98 99 100 101 102 103 104 105 106 107 108 109 110 111 112 113 114 115 116 117 118 119 120 121 122 123 124 125 126 127 128 129 130 131 132 133 134 135 136 137 138 139 140 141 142 143 144 145 146 147 148 149 150 151 152 153 154 155 156 157 158 159 160 161 162 163 164 165 166 167 168 169 170 171 172 173 174 175 176 177 178 179 180 181 182 183 184 185 186 187 188 189 190 191 192 193 194 195 196 197 198 199 200 201 202 203 204 205 206 207 208 209 210 211 212 213 214 215 216 217 218 219 220 221 222 223 224 225 226 227 228 229 230 231 232 233 234 235 236 237 238 239 240 241 242 243 244 245 246 247 248 249 250 251 252 253 254 255 256 257 258 259 260 261 262 263 264 265 266 267 268 269 270 271 272 273 274 275 276 277 278 279 280 281 282 283 284 285 286 287 288 289 290 291 292 293 294 295 296 297 298 299 300 301 302 303 304 305 306 307 308 309 310 311 312 313 314 315 316 317 318 319 320 321 322 323 324 325 326 327 328 329 330 331 332 333 334 335 336 337 338 339 340 341 342 343 344 345 346 347 348 349 350 351 352 353 354 355 356 357 358 359 360 361 362 363 364 365 366 367 368 369 370 371 372 373 374 375 376 377 378 379 380 381 382 383 384 385 386 387 388 389 390 391 392 393 394 395 396 397 398 399 400 401 402 403 404 405 406 407 408 409 410 411 412 413 414 415 416 417 418 419 420 421 422 423 424 425 426 427 428 429 430 431 432 433 434 435 436 437 438 439 440 441 442 443 444 445 446 447 448 449 450 451 452 453 454 455 456 457 458 | 1 1 1         10780 25714 25714 4104   21610                   217                                           160 20 20 147 147       147 147   20           480 646 646 646   646           480 356 356   350   6       376   1104 1104 43                       4000 4000 15483                     4000 15483 15483 15483   5153   15483 15483 15483 14556   927             1601 1601 5498 5498 31059     31059 31059   29340   31059 1719     3779       1140 1140 14052 14052 15582     15582 15582   13450   2132 2132   602                   160 160   160     160 469 469 469 471 471   457   14 14 14   12 12   2   469 457   12     12             12                                               2340 2340 2340     2917   2917       6399 6399 6399 6399     6399 6399   3427       2972           2972 1922 1922   542     1050           2972               2972     1380 1380 7872 6492             1380       1380         1341 1341                 2972 2469       2972 2301                 2917 508       2409     528 528           528       2409                         5440 16998 1169       5300 18800 2744                                                                         1 1 5336 5336   5336     5336                 5336       4     1   1 4     1 3 2741     2741         1   1 3 | ``` (function () { 	var $P = Date.Parsing; 	var _ = $P.Operators = { 		// 		// Tokenizers 		// 		rtoken: function (r) { // regex token 			return function (s) { 				var mx = s.match(r); 				if (mx) { 					return ([ mx[0], s.substring(mx[0].length) ]); 				} else { 					throw new $P.Exception(s); 				} 			}; 		}, 		token: function () { // whitespace-eating token 			return function (s) { 				return _.rtoken(new RegExp("^\\s*" + s + "\\s*"))(s); 			}; 		}, 		stoken: function (s) { // string token 			return _.rtoken(new RegExp("^" + s)); 		},   		// Atomic Operators   		until: function (p) { 			return function (s) { 				var qx = [], rx = null; 				while (s.length) { 					try { 						rx = p.call(this, s); 					} catch (e) { 						qx.push(rx[0]); 						s = rx[1]; 						continue; 					} 					break; 				} 				return [ qx, s ]; 			}; 		}, 		many: function (p) { 			return function (s) { 				var rx = [], r = null; 				while (s.length) { 					try { 						r = p.call(this, s); 					} catch (e) { 						return [ rx, s ]; 					} 					rx.push(r[0]); 					s = r[1]; 				} 				return [ rx, s ]; 			}; 		},   		// generator operators -- see below 		optional: function (p) { 			return function (s) { 				var r = null; 				try { 					r = p.call(this, s); 				} catch (e) { 					return [ null, s ]; 				} 				return [ r[0], r[1] ]; 			}; 		}, 		not: function (p) { 			return function (s) { 				try { 					p.call(this, s); 				} catch (e) { 					return [null, s]; 				} 				throw new $P.Exception(s); 			}; 		}, 		ignore: function (p) { 			return p ? 			function (s) { 				var r = null; 				r = p.call(this, s); 				return [null, r[1]]; 			} : null; 		}, 		product: function () { 			var px = arguments[0], 			qx = Array.prototype.slice.call(arguments, 1), rx = []; 			for (var i = 0 ; i < px.length ; i++) { 				rx.push(_.each(px[i], qx)); 			} 			return rx; 		}, 		cache: function (rule) { 			var cache = {}, cache_length = 0, cache_keys = [], CACHE_MAX = Date.Config.CACHE_MAX \|\| 100000, r = null; 			var cacheCheck = function () { 				Iif (cache_length === CACHE_MAX) { 					// kill several keys, don't want to have to do this all the time... 					for (var i=0; i < 10; i++) { 						var key = cache_keys.shift(); 						if (key) { 							delete cache[key]; 							cache_length--; 						} 					} 				} 			}; 			return function (s) { 				cacheCheck(); 				try { 					r = cache[s] = (cache[s] \|\| rule.call(this, s)); 				} catch (e) { 					r = cache[s] = e; 				} 				cache_length++; 				cache_keys.push(s); 				if (r instanceof $P.Exception) { 					throw r; 				} else { 					return r; 				} 			}; 		},   		// vector operators -- see below 		any: function () { 			var px = arguments; 			return function (s) { 				var r = null; 				for (var i = 0; i < px.length; i++) { 					Iif (px[i] == null) { 						continue; 					} 					try { 						r = (px[i].call(this, s)); 					} catch (e) { 						r = null; 					} 					if (r) { 						return r; 					} 				} 				throw new $P.Exception(s); 			}; 		}, 		each: function () { 			var px = arguments; 			return function (s) { 				var rx = [], r = null; 				for (var i = 0; i < px.length ; i++) { 					Iif (px[i] == null) { 						continue; 					} 					try { 						r = (px[i].call(this, s)); 					} catch (e) { 						throw new $P.Exception(s); 					} 					rx.push(r[0]); 					s = r[1]; 				} 				return [ rx, s]; 			}; 		}, 		all: function () { 			var px = arguments, _ = _; 			return _.each(_.optional(px)); 		},   		// delimited operators 		sequence: function (px, d, c) { 			d = d \|\| _.rtoken(/^\s*/); 			c = c \|\| null; 			 			Iif (px.length === 1) { 				return px[0]; 			} 			return function (s) { 				var r = null, q = null; 				var rx = []; 				for (var i = 0; i < px.length ; i++) { 					try { 						r = px[i].call(this, s); 					} catch (e) { 						break; 					} 					rx.push(r[0]); 					try { 						q = d.call(this, r[1]); 					} catch (ex) { 						q = null; 						break; 					} 					s = q[1]; 				} 				if (!r) { 					throw new $P.Exception(s); 				} 				Iif (q) { 					throw new $P.Exception(q[1]); 				} 				Iif (c) { 					try { 						r = c.call(this, r[1]); 					} catch (ey) { 						throw new $P.Exception(r[1]); 					} 				} 				return [ rx, (r?r[1]:s) ]; 			}; 		},   		// 		// Composite Operators 		//   		between: function (d1, p, d2) { 			d2 = d2 \|\| d1; 			var _fn = _.each(_.ignore(d1), p, _.ignore(d2)); 			return function (s) { 				var rx = _fn.call(this, s); 				return [[rx[0][0], r[0][2]], rx[1]]; 			}; 		}, 		list: function (p, d, c) { 			d = d \|\| _.rtoken(/^\s*/); 			c = c \|\| null; 			return (p instanceof Array ? 				_.each(_.product(p.slice(0, -1), _.ignore(d)), p.slice(-1), _.ignore(c)) : 				_.each(_.many(_.each(p, _.ignore(d))), px, _.ignore(c))); 		}, 		set: function (px, d, c) { 			d = d \|\| _.rtoken(/^\s*/); 			c = c \|\| null; 			return function (s) { 				// r is the current match, best the current 'best' match 				// which means it parsed the most amount of input 				var r = null, p = null, q = null, rx = null, best = [[], s], last = false; 				// go through the rules in the given set 				for (var i = 0; i < px.length ; i++) {   					// last is a flag indicating whether this must be the last element 					// if there is only 1 element, then it MUST be the last one 					q = null; 					p = null; 					r = null; 					last = (px.length === 1); 					// first, we try simply to match the current pattern 					// if not, try the next pattern 					try { 						r = px[i].call(this, s); 					} catch (e) { 						continue; 					} 					// since we are matching against a set of elements, the first 					// thing to do is to add r[0] to matched elements 					rx = [[r[0]], r[1]]; 					// if we matched and there is still input to parse and  					// we don't already know this is the last element, 					// we're going to next check for the delimiter ... 					// if there's none, or if there's no input left to parse 					// than this must be the last element after all ... 					if (r[1].length > 0 && ! last) { 						try { 							q = d.call(this, r[1]); 						} catch (ex) { 							last = true; 						} 					} else { 						last = true; 					}   					// if we parsed the delimiter and now there's no more input, 					// that means we shouldn't have parsed the delimiter at all 					// so don't update r and mark this as the last element ... 					Iif (!last && q[1].length === 0) { 						last = true; 					}     					// so, if this isn't the last element, we're going to see if 					// we can get any more matches from the remaining (unmatched) 					// elements ... 					if (!last) { 						// build a list of the remaining rules we can match against, 						// i.e., all but the one we just matched against 						var qx = []; 						for (var j = 0; j < px.length ; j++) { 							if (i !== j) { 								qx.push(px[j]); 							} 						}   						// now invoke recursively set with the remaining input 						// note that we don't include the closing delimiter ... 						// we'll check for that ourselves at the end 						p = _.set(qx, d).call(this, q[1]);   						// if we got a non-empty set as a result ... 						// (otw rx already contains everything we want to match) 						if (p[0].length > 0) { 							// update current result, which is stored in rx ... 							// basically, pick up the remaining text from p[1] 							// and concat the result from p[0] so that we don't 							// get endless nesting ... 							rx[0] = rx[0].concat(p[0]); 							rx[1] = p[1]; 						} 					}   					// at this point, rx either contains the last matched element 					// or the entire matched set that starts with this element.   					// now we just check to see if this variation is better than 					// our best so far, in terms of how much of the input is parsed 					if (rx[1].length < best[1].length) { 						best = rx; 					}   					// if we've parsed all the input, then we're finished 					if (best[1].length === 0) { 						break; 					} 				}   				// so now we've either gone through all the patterns trying them 				// as the initial match; or we found one that parsed the entire 				// input string ...   				// if best has no matches, just return empty set ... 				if (best[0].length === 0) { 					return best; 				}   				// if a closing delimiter is provided, then we have to check it also 				if (c) { 					// we try this even if there is no remaining input because the pattern 					// may well be optional or match empty input ... 					try { 						q = c.call(this, best[1]); 					} catch (ey) { 						throw new $P.Exception(best[1]); 					}   					// it parsed ... be sure to update the best match remaining input 					best[1] = q[1]; 				} 				// if we're here, either there was no closing delimiter or we parsed it 				// so now we have the best match; just return it! 				return best; 			}; 		}, 		forward: function (gr, fname) { 			return function (s) { 				return gr[fname].call(this, s); 			}; 		},   		// 		// Translation Operators 		// 		replace: function (rule, repl) { 			return function (s) { 				var r = rule.call(this, s); 				return [repl, r[1]]; 			}; 		}, 		process: function (rule, fn) { 			return function (s) { 				var r = rule.call(this, s); 				return [fn.call(this, r[0]), r[1]]; 			}; 		}, 		min: function (min, rule) { 			return function (s) { 				var rx = rule.call(this, s); 				if (rx[0].length < min) { 					throw new $P.Exception(s); 				} 				return rx; 			}; 		} 	}; 	   	// Generator Operators And Vector Operators   	// Generators are operators that have a signature of F(R) => R, 	// taking a given rule and returning another rule, such as  	// ignore, which parses a given rule and throws away the result.   	// Vector operators are those that have a signature of F(R1,R2,...) => R, 	// take a list of rules and returning a new rule, such as each.   	// Generator operators are converted (via the following _generator 	// function) into functions that can also take a list or array of rules 	// and return an array of new rules as though the function had been 	// called on each rule in turn (which is what actually happens).   	// This allows generators to be used with vector operators more easily. 	// Example: 	// each(ignore(foo, bar)) instead of each(ignore(foo), ignore(bar))   	// This also turns generators into vector operators, which allows 	// constructs like: 	// not(cache(foo, bar)) 	 	var _generator = function (op) { 		function gen() { 			var args = null, rx = [], px, i; 			Iif (arguments.length > 1) { 				args = Array.prototype.slice.call(arguments); 			} else Iif (arguments[0] instanceof Array) { 				args = arguments[0]; 			} 			Iif (args) { 				px = args.shift(); 				if (px.length > 0) { 					args.unshift(px[i]); 					rx.push(op.apply(null, args)); 					args.shift(); 					return rx; 				} 			} else { 				return op.apply(null, arguments); 			} 		}   		return gen; 	}; 	 	var gx = "optional not ignore cache".split(/\s/); 	 	for (var i = 0 ; i < gx.length ; i++) { 		_[gx[i]] = _generator(_[gx[i]]); 	}   	var _vector = function (op) { 		return function () { 			Iif (arguments[0] instanceof Array) { 				return op.apply(null, arguments[0]); 			} else { 				return op.apply(null, arguments); 			} 		}; 	}; 	 	var vx = "each any all".split(/\s/); 	 	for (var j = 0 ; j < vx.length ; j++) { 		_[vx[j]] = _vector(_[vx[j]]); 	} 	 }()); ``` |
```

Generated by istanbul at Sun Nov 16 2014 12:08:33 GMT-0800 (PST)
